# Enterovirus D68 Infection in Human Primary Airway and Brain Organoids: No Additional Role for Heparan Sulfate Binding for Neurotropism

**DOI:** 10.1128/spectrum.01694-22

**Published:** 2022-09-26

**Authors:** Adithya Sridhar, Josse A. Depla, Lance A. Mulder, Eveliina Karelehto, Lieke Brouwer, Leonie Kruiswijk, Renata Vieira de Sá, Adam Meijer, Melvin M. Evers, Frank J. M. van Kuppeveld, Dasja Pajkrt, Katja C. Wolthers

**Affiliations:** a Amsterdam UMC, University of Amsterdam, Amsterdam Institute for Infection and Immunity, Department of Medical Microbiology, OrganoVIR Labs, Amsterdam, The Netherlands; b Amsterdam UMC, University of Amsterdam, Vrije Universiteit, Emma Children’s Hospital Department of Pediatric Infectious Diseases, Amsterdam, The Netherlands; c uniQure Biopharma B.V., Amsterdam, The Netherlands; d National Institute for Public Health and Environment, Centre for Infectious Diseases Research and Laboratory Surveillance, Bilthoven, The Netherlands; e Virology Division, Department of Infectious Diseases and Immunology, Faculty of Veterinary Medicine, Utrecht University, Utrecht, The Netherlands; Wayne State University

**Keywords:** receptors, cerebral organoids, enterovirus, enterovirus D68, human airway epithelial cultures (HAE), neurotropism

## Abstract

Enterovirus D68 (EV-D68) is an RNA virus that can cause outbreaks of acute flaccid paralysis (AFP), a polio-like disease. Before 2010, EV-D68 was a rare pathogen associated with mild respiratory symptoms, but the recent EV-D68 related increase in severe respiratory illness and outbreaks of AFP is not yet understood. An explanation for the rise in severe disease is that it may be due to changes in the viral genome resulting in neurotropism. In this regard, in addition to sialic acid, binding to heparan sulfate proteoglycans (HSPGs) has been identified as a feature for viral entry of some EV-D68 strains in cell lines. Studies in human primary organotypic cultures that recapitulate human physiology will address the relevance of these HSPG-binding mutations for EV-D68 infection in vivo. Therefore, in this work, we studied the replication and neurotropism of previously determined sialic acid-dependent and HSPG-dependent strains using primary human airway epithelial (HAE) cultures and induced human pluripotent stem cell (iPSC)-derived brain organoids. All three strains (B2/2042, B2/947, and A1/1348) used in this study infected HAE cultures and human brain organoids (shown for the first time). Receptor-blocking experiments in both cultures confirm that B2/2042 infection is solely dependent on sialic acid, while B2/947 and A1/1348 (HSPG to a lesser extent) binds to sialic acid and HSPG for cell entry. Our data suggest that HSPG-binding can be used by EV-D68 for entry in human physiological models but offers no advantage for EV-D68 infection of brain cells.

**IMPORTANCE** Recent outbreaks of enterovirus D68, a nonpolio enterovirus, is associated with a serious neurological condition in young children, acute flaccid myelitis (AFM). As there is no antiviral treatment or vaccine available for EV-D68 it is important to better understand how EV-D68 causes AFM and why only recent outbreaks are associated with AFM. We investigated if a change in receptor usage of EV-D68 increases the virulence of EV-D68 in the airway or the central nervous system and thus could explain the increase in AFM cases. We studied this using physiologically relevant human airway epithelium and cerebral organoid cultures that are physiologically relevant human models. Our data suggest that heparan sulfate proteoglycans can be used by EV-D68 as an additional entry receptor in human physiological models but offers no advantage for EV-D68 infection of brain cells, and our data show the potential of these 46 innovative models for virology.

## INTRODUCTION

The human enterovirus D68 (EV-D68) is a single-stranded positive-sense RNA virus from the *Enterovirus* (EV) genus in the *Picornaviridae* family. EV-D68 was first isolated in 1962 in California from children with respiratory infections (1). It remained a rare pathogen that caused mild respiratory disease but since 2010 there has been an increased association with severe respiratory illness, and since 2014, outbreaks of acute flaccid paralysis (AFP) have been linked to EV-D68 infection (2, 3). With the near global eradication of poliomyelitis, emergence of AFP with clinical resemblance to poliomyelitis caused by other nonpolio enteroviruses is a serious concern (4).

The reasons behind this increase in pathogenicity of EV-D68 at both the airway (primary site for entry and replication) and central nervous system (CNS, secondary site of infection) is not clear. Increased pathogenicity could be due to a general increase in virulence leading to more severe infections and higher rates of neurological complications (5). Contemporary strains might have acquired the ability to bind different or additional receptors that enable a broader cell tropism or more efficient infection of the primary and secondary infection sites. Indeed, in addition to α2,3- and α2,6-linked sialic acids, some contemporary strains are also capable of cell entry by binding ICAM-5 or sulfated glycosaminoglycans (sGAGs), which may play a role in increased virulence (6–10). However, since ICAM-5 is not expressed in the human respiratory tract or spinal cord, the role of ICAM-5 in increasing EV-D68 virulence is disputed (5).

Cell surface endocytosis receptors such as sGAGs and heparan sulfate proteoglycans (HSPGs), in particular, are interesting as HSPG is known to play a role in binding of other neurotropic viruses such as herpes simplex virus, dengue virus, and some echoviruses (11–15). Moreover, HSPG-dependence through an intrahost adaptation resulting in neurovirulence has been demonstrated for enterovirus A71 (EV-A71) (16, 17). The HSPG-binding mutation (L97R in the VP1 region) was not present in EV-A71 respiratory isolates but only found in isolates from feces, blood, and cerebrospinal fluid. This HSPG-binding mutation was also shown to modulate *ex vivo* tissue tropism and was concluded to promote viral dissemination and neurotropism of EV-A71 (16, 17). In a similar manner, HSPG-binding mutations could promote virulence and neurotropism of EV-D68.

Although the HSPG-binding mutation in EV-D68 (E271K in the VP1 region) was initially discovered through a cell culture adaptation, the HSPG-binding mutation at this position is also found in some clinical isolates (7, 18–21). Moreover, work performed on a neuroblastoma cell line showed an increased replication and attachment of EV-D68 HSPG-binding variants. Evaluating the use of these receptors using human organotypic cultures will provide more insight into *in vivo* pathogenesis as these complex cultures have better value for translational studies over traditionally used cell lines (22). The primary replication site (airway) of EV-D68 infection can be modeled using human airway epithelial cultures that closely mimic *in vivo* tissue in respect to multicellular composition, epithelial function, and innate immune responses and have been extensively used in virology for pathogenesis and antiviral studies (23–27). The secondary replication site can be modeled using human-induced pluripotent stem cell (iPSC)-derived cerebral organoids that closely resemble the developing human brain and have been used for studying neurotropic viruses (28, 29). We, and others, previously characterized that these iPSC-derived cerebral organoids contain various brain cells, including neural progenitor cells, neurons, and astrocytes (30, 31). Therefore, in this study, we assessed the roles of sialic acid and HSPG for the infection of HAE and cerebral organoid cultures with three different contemporary EV-D68 strains that use different receptors for entry in cell lines (B2/2042, solely dependent on sialic acid for entry, and B2/947 and A1/1348 using sialic acid and another nonsialic acid receptor for entry) (10).

## RESULTS

### EV-D68 replicates efficiently in human airway epithelial (HAE) cultures.

**FIG 1:**
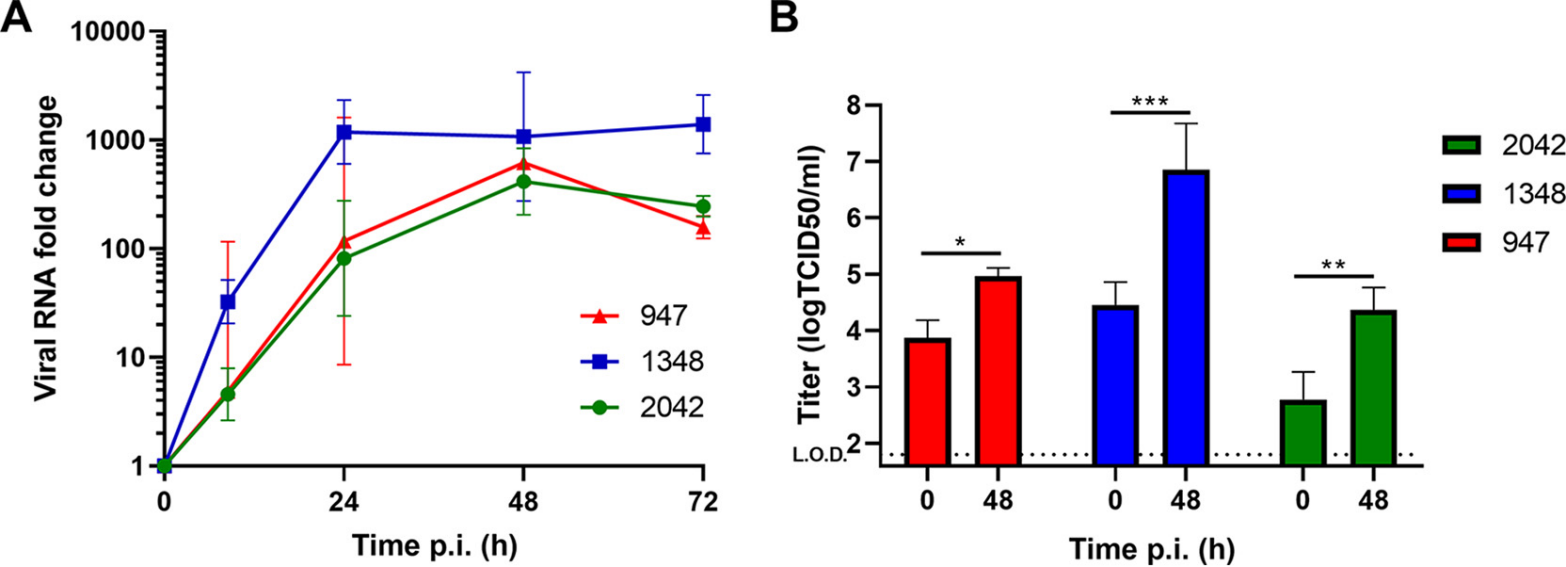
Replication of different EV-D68 strains in human airway epithelial (HAE) cultures. (A) Growth curves for EV-D68 strains in the HAE model. Viral RNA was measured by RT-qPCR analysis of the culture medium at specified time points. (B) Replication competent viral titers were measured using a medium tissue culture infectious dose 50 (TCID50) assay. The geometric mean and standard deviation of three biological replicates are shown in both panels. In panel B, *P* values were calculated by Sidak’s multiple-comparison test. (*, *P* < 0.05; **, *P* < 0.01; ***, *P* > 0.001; *n* = 3 biological replicates). For mock-infected HAE cultures, no increase in viral RNA was measured.

The replication of one sialic acid-dependent strain (B2/2042) and two (B2/947 and A1/1348) strains using sialic acid and one other receptor was tested by apical infection of HAE cultures derived from three different donors (9, 10). We observed that all three strains were able to replicate in HAE cultures (Fig. 1A) and the increase over 48 h postinfection (hpi) was statistically significant, as measured by reverse transcription quantitative PCR (RT-qPCR), while no viral RNA was measured in mock-infected organoids. Tissue culture infectious dose 50 (TCID50) analyses demonstrated that EV-D68 infection resulted in significant production of infectious viral particles at 48 hpi of all three strains (Fig. 1B). Based on the TCID50 data, the viral release was seen primarily on the apical side with undetectable levels on the basolateral compartment of the HAE cultures (data not shown).

### EV-D68 can use HSPG as a second receptor in HAE cultures.

**FIG 2:**
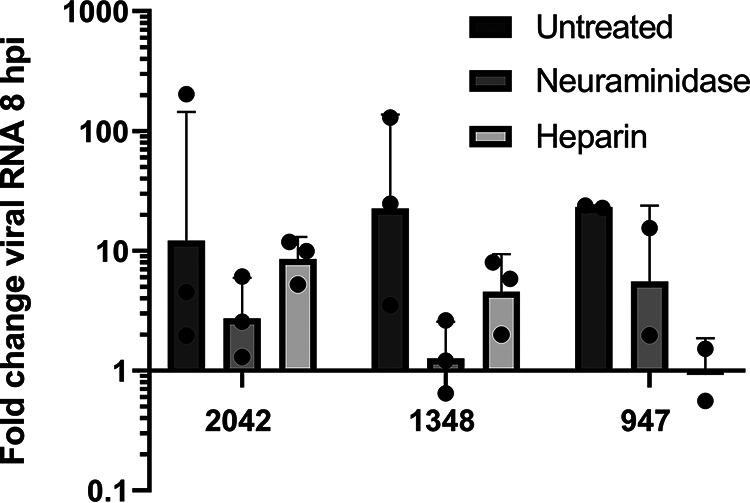
Receptor usage of different EV-D68 strains in HAE cultures. The fold change in viral RNA copies at 8 hpi, normalized to 0 hpi, in medium of EV-D68 infected HAE cultures where the binding to sialic acid receptor was blocked by neuraminidase and binding to HSPG was blocked using soluble heparin, while untreated HAE were infected with EV-D68 without either treatment. Viral RNA was measured by RT-qPCR analysis of the culture medium at 0 and 8 hpi. For B2/2042 and A1/1348; the results are based on three biological replicates. For B2/947 two biological replicates are shown as the replication cycle was too slow in the third donor, and therefore no replication was yet observed after 8 hpi based on the replication curves in Fig. 1. For mock-infected HAE cultures, no increase in viral RNA was measured.

Sialic acid-dependence in human airway was tested by pretreating the HAE cultures, prior to viral infection, with neuraminidase which hydrolyzes α2,3-, α2,6-, and α2,8-linked sialic acids on the cell surface. HSPG-dependence was tested by pretreating the virus, prior to inoculation of HAE, with soluble heparin to block the heparan sulfate (HS) binding sites on the viral capsid. Neuraminidase treatment to remove sialic acid resulted in fewer viral RNA at 8 hpi for all three EV-D68 strains, confirming that all strains can use sialic acid for entry (Fig. 2). Heparin treatment of the three strains of EV-D68 resulted in inhibition of B2/947 and to a lesser extent of A1/1348 while having no effect on B2/2042.

### EV-D68 infection depletes ciliated cells in the human airway epithelium.

**FIG 3:**
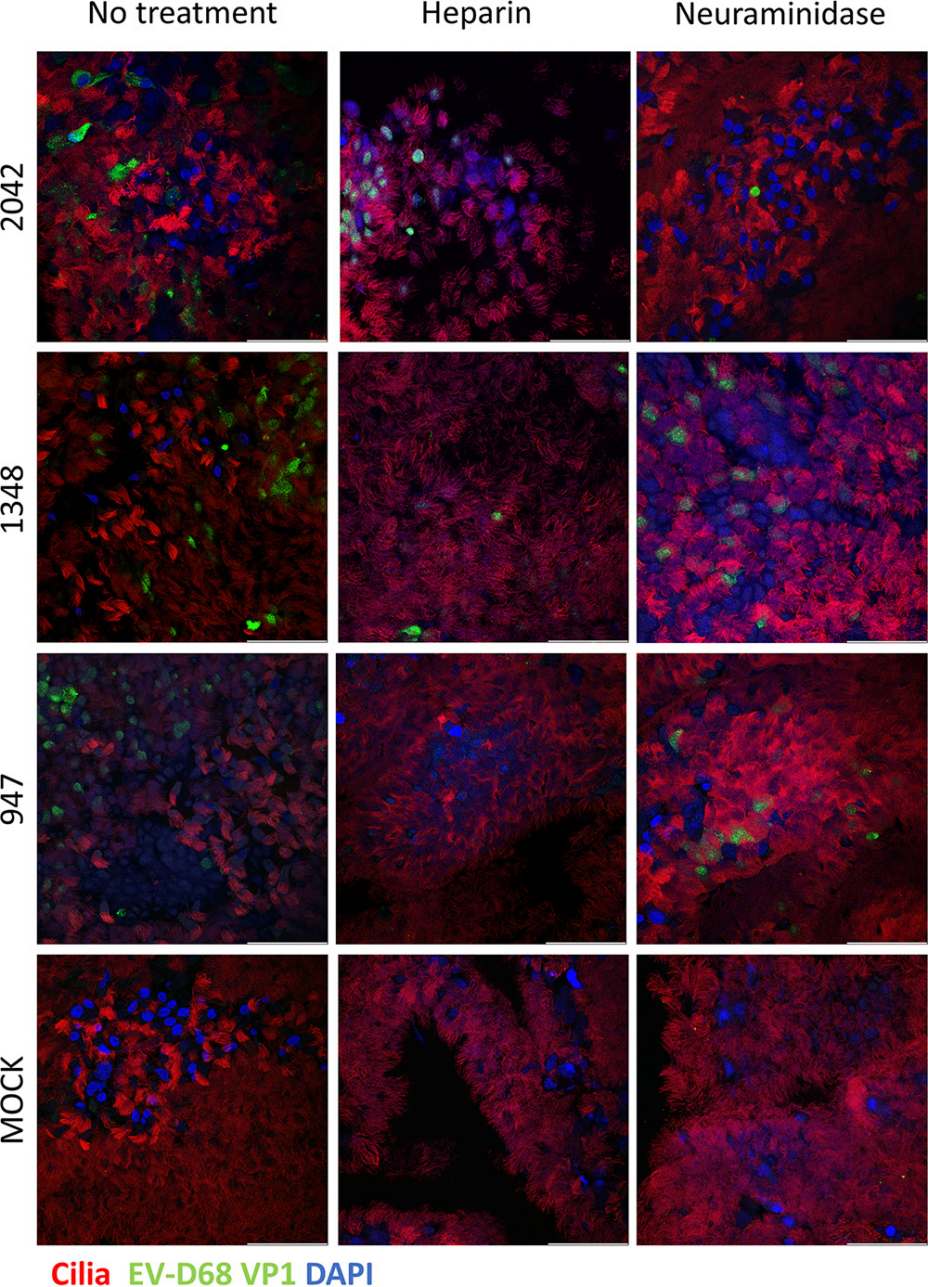
Immunofluorescent staining of EV-D68 infection of HAE cultures 8 hpi. Immunofluorescent staining of EV-D68 infection of HAE cultures 8 hpi under different conditions. EV-D68 (green) is colocalized in ciliated cells (red). The basal cells were present in a different plane and remained uninfected. A 3D side view of the confocal z-stack is shown in Fig. S3. Scale bars, 50 μm.

**FIG 4:**
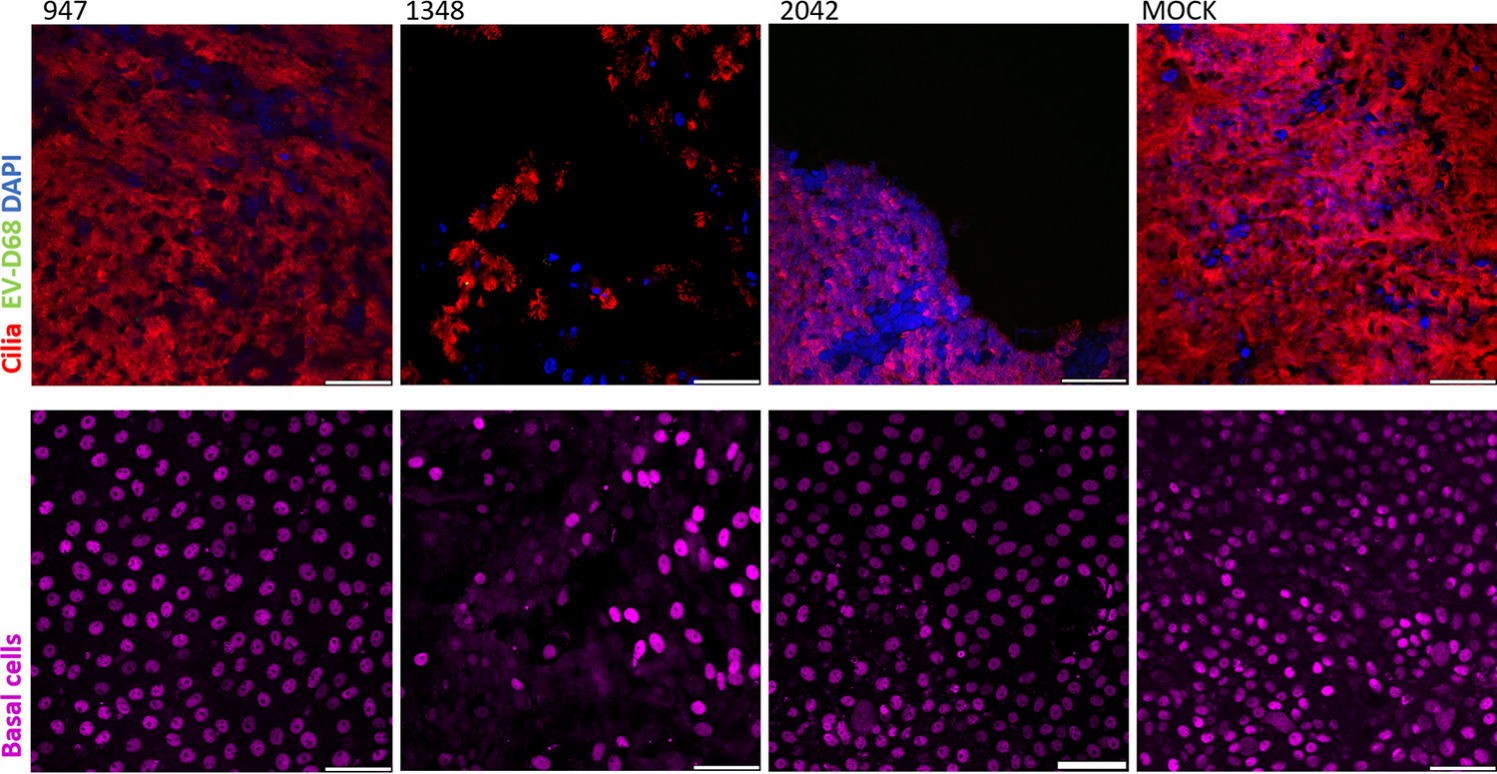
Immunofluorescent staining of EV-D68 infection of HAE cultures 72 hpi. EV-D68 A1/1348 and B2/2042 infection disrupts cell monolayer as seen by depletion of infected ciliated cells (red) on the apical side (top row). No EV-D68 staining (green) was seen at 72 hpi likely due to death of susceptible cells which was consistent with the growth curves in Fig. 1A. The basal cells (purple) were in a different plane (bottom row) and remained unaffected. Scale bars, 50 μm.

To determine the cell tropism of EV-D68, we performed imaging with immunofluorescent staining and confocal microscopy of the HAE cultures. The cultures were fixed and tripled stained for ciliated cell marker β-tubulin, basal cell marker p63, and EV-D68 VP1 protein or triple stained for secretory cell marker MUC5B, basal cell marker p63, and EV-D68 VP1 protein. At 8 hpi, viral staining was colocalized in ciliated cells (Fig. 3). Neuraminidase treatment of the HAE cultures resulted in fewer EV-D68 B2/2042 virus-positive cells, while heparin treatment of EV-D68 resulted in fewer virus-positive cells of the B2/947 and A1/1348 strains. Confocal imaging of the stained HAE inserts at 72 hpi revealed that EV-D68 A1/1348 and B2/2042 infection resulted in a depletion of ciliated cells, while the ciliated cells remained intact in EV-D68 B2/947 and MOCK infected cultures (Fig. 4). However, colocalization of VP1-staining could not be observed at 72 h postinfection. HAE infection with the three EV-D68 strains did not affect the basal cells and no colocalization of VP1 staining with p63 staining was observed. Although the HAE cultures had good mucus coverage on the cell surface, MUC5B staining of secretory cells was inconclusive and MUC5B positive cells were not observed in most of the cultures.

### EV-D68 infects human iPSC-derived cerebral organoids.

**FIG 5:**
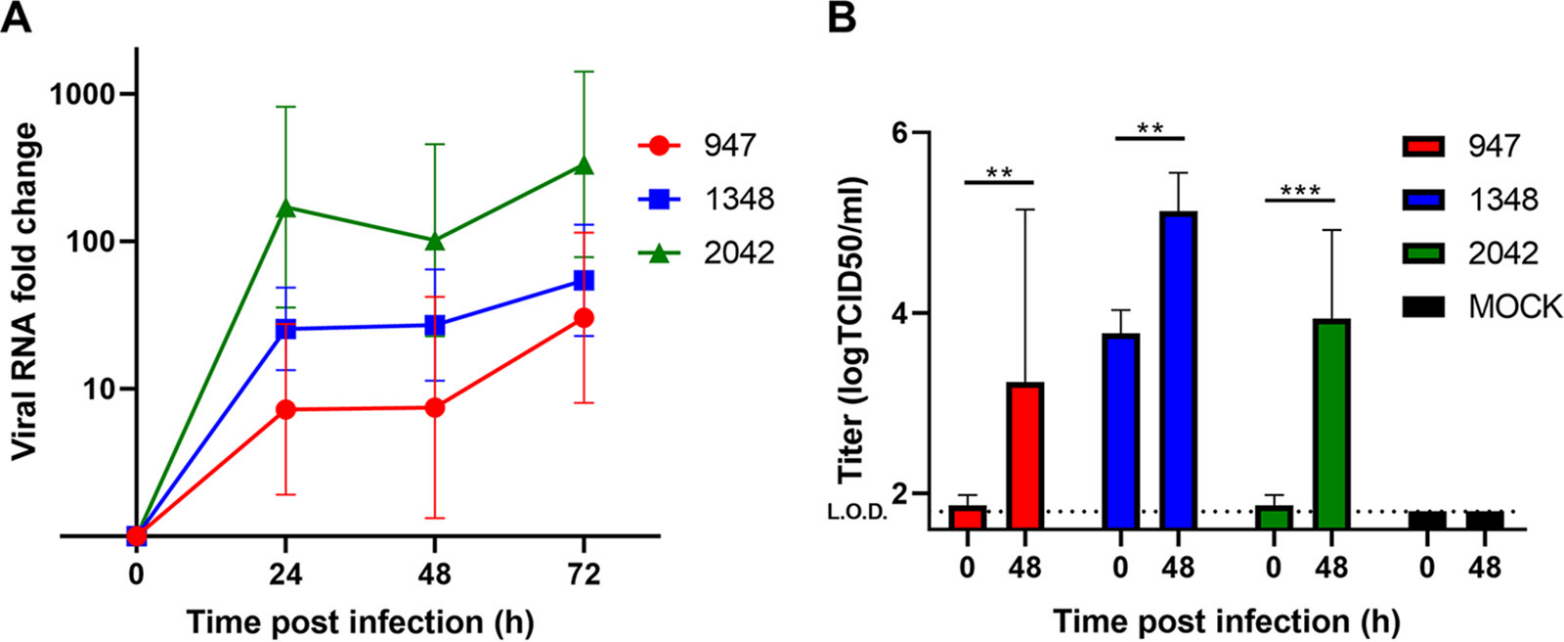
Replication of different EV-D68 strains in cerebral organoids. (A) Growth curves for EV-D68 strains in cerebral organoids. Viral RNA was measured by RT-qPCR analysis of the culture medium at specified time points. (*n* = 4 batches) (B) Replication competent viral titers were measured using a medium tissue culture infectious dose (TCID50) assay. (Limit of detection [L.O.D.], *n* = 1 batch of 5 organoids). The geometric mean and 95% confidence interval are shown in both panels. *P* values were calculated by Sidak’s multiple-comparison test. (**, *P* < 0.01; ***, *P* > 0.001).

To assess whether EV-D68 is able to infect cerebral organoids and if the infection is dependent on HSPG-binding, we infected four different batches of 45- to 52-day-old cerebral organoids with the three EV-D68 strains (B2/2042, B2/947, and A1/1348). We observed that all three strains were able to infect the cerebral organoids as an increase in viral RNA was measured in the medium of infected cerebral organoids and not in mock-infected organoids by RT-qPCR over time (Fig. 5A). TCID50 analyses showed a significant increase in infectious virus for all strains in the medium at 48 hpi and not in mock-infected organoids (Fig. 5B).

### EV-D68 can use HSPG as a second receptor in cerebral organoids.

**FIG 6:**
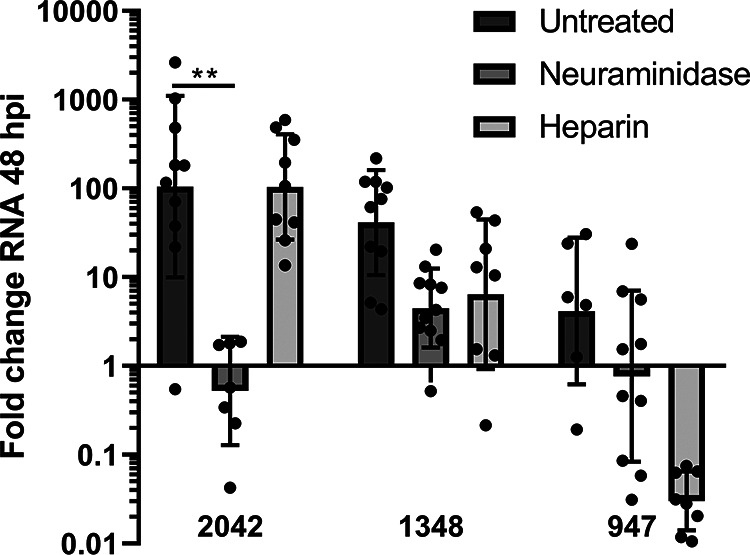
Receptor use of different EV-D68 strains in cerebral organoids. The fold change in viral RNA copies in medium of EV-D68 infected cerebral organoids where the binding to sialic acid was blocked by neuraminidase and binding to HSPG by soluble heparin, while untreated cerebral organoids were infected with EV-D68 without neuraminidase or heparin treatment. Viral RNA was measured by RT-qPCR analysis of the culture medium at 0 and 48 hpi, fold change was calculated by normalizing viral to t0 (each dot represents an individual organoid originating from 3 batches of organoids). Geometric mean and geometric standard deviation are shown. *P* values were calculated by Sidak’s multiple-comparison test (**, *P* < 0.01).

Next, we investigated the receptor usage of the different strains in brain organoids by either pretreating brain organoids with neuraminidase or pretreating the virus, prior to inoculation, with soluble heparin to block HSPG-binding sites on the viral capsid. In line with the results in HAE, B2/2042 replication was dependent on sialic acid, as neuraminidase pretreatment reduced the viral fold change significantly (*P* > 0.01), while heparin treatment did not inhibit replication (Fig. 6). For the other two strains, blocking of sialic acid as an entry receptor also inhibited replication, but not significantly. EV-D68 strain A1/1348 was similarly inhibited by blocking of HSPG as sialic acid removal, while the EV-D68 strain B2/947 was most strongly inhibited by blocking of HSPG. However, due to high variation in fold changes between each cerebral organoid, no significant reduction of viral replication was observed.

## DISCUSSION

In this study, we provide insights into EV-D68 receptor usage in primary organotypic cell culture models. Given the importance of various cell entry receptors for picornavirus tropism, we evaluated if HSPG-binding as previously identified using cell lines confer any benefits in human primary cell culture. This HSPG-binding mutation (E271K in VP1 of EV-D68) was particularly interesting as similar HSPG-binding mutations (L97R in VP1 of EV-A71) have been shown to promote viral dissemination and neurotropism for another enterovirus, EV-A71 (16, 17). Moreover, as HSPG are ubiquitously expressed across most tissues in humans, our hypothesis was that the ability to bind HSPG could broaden the tropism for EV-D68.

Specific characteristics of HSPG and other sGAGs as having a net negative charge and interacting electrostatically with a virus are well defined (32, 33). However, the role of HSPG-binding and increased virulence is controversial (11). HSPG-binding is often described as a result of cell culture adaptation that enhances viral infection *in vitro* (34). For respiratory viruses, the ability to bind HSPG and other sGAGs is likely to be detrimental as the airway mucus is abundant with sGAGs (35). Moreover, we did not see any differences in cell tropism between HSPG-binding and non HSPG-binding strains in the airway. Although HSPG is diffusely expressed throughout HAE cultures, both types of strains only infected the ciliated cells consistent with EV-D68 infection of airway cultures (23, 36). This alone does not preclude the usefulness of HSPG-binding for EV-D68 *in vivo*. We did not study if the HSPG-binding mutation of EV-D68 alters the immune response upon infection and altered immune responses could influence immune evasion. In a cell line the ability to bind sGAGs was already shown to be beneficial for EV-D68 B2/947 infection. By binding sGAGs, EV-D68 could bypass PLA2G16 facilitated delivery of viral RNA into the cytoplasm which could potentially alter downstream immune responses (9). Thus, it would be interesting to see if any alterations to innate immune response arises due to HSPG-binding in primary cell cultures.

HSPG-binding mutations may also be relevant for *in vivo* pathogenesis even if they do not necessarily confer benefits at the primary replication site. As seen with EV-A71, an intrahost emergence of HSPG-binding mutants might confer neurovirulence. Therefore, we evaluated CNS infection of the different EV-D68 strains using cerebral organoids. Consistent with our findings in the HAE cultures, all three strains were able to infect cerebral organoids. Contrary to our initial expectation, HSPG-binding did not appear to promote neurotropism of EV-D68 and some EV-D68 strains could use HSPG-binding for infection of cerebral organoids. For this study, we employed human cerebral organoids as an infection model for EV-D68 infection of the CNS in humans and show that EV-D68 can infect the brain. However, it is important to note that currently there is no evidence that EV-D68 infects brain cells *in vivo*. The next step in this research would be to evaluate EV-D68 infectivity in recently developed human iPSC-derived motor neuron organoids, spinal cord organoids, or cortico-motor assembloids as models for EV-D68 induced AFP (37–40).

In our study, we tested the possibility that HSPG-binding would result in neurovirulence of EV-D68. It is possible that EV-D68 strains have other mutations that result in a phenotype with a broader cell tropism (41, 42). For instance, contemporary strains have a contraction of the spacer in the 5′ untranslated region of the viral RNA. This specific site is an established region of virulence in poliovirus and other picornaviruses (43–45). However, both contemporary and pre-outbreak isolates of EV-D68 can replicate in neuronal cell lines as well in primary neurons and astrocytes derived from human pluripotent stem cells (hPSCs) indicating that this contraction of the spacer might not be responsible for increased virulence (46). Another possibility is the ability of contemporary strains to replicate (*in vitro* on cell lines) at equal efficiency at 32°C or 37°C, while the replication of prototype strains such as the EV-D68 Fermon strain (isolated from a respiratory sample in 1962) is attenuated at higher temperatures (47). It would be worthwhile to verify these findings using complex 3D CNS models, such as motor neuron or spinal cord models.

In conclusion, in our experiments we did not obtain evidence that usage of HSPG as an additional receptor by EV-D68 has additional benefits in physiological human models of the primary and secondary replication sites. It is possible that HSPG-binding is a cell culture adaptation and is most likely not the reason for the sudden emergence or neurotropism of contemporary EV-D68 strains (10). Further research using clinical EV-D68 isolates from AFP affected patients and novel human 3D CNS (motor neuron or spinal cord) models is necessary to exclude the role of HSPG and sGAGs for EV-D68 neuroinfection *in vivo*.

## MATERIALS AND METHODS

### Viruses and cells.

EV-D68 strains B2/2042 (4310902042, isolated from a 1-year-old male diagnosed with bronchiolitis), B2/947 (4310900947, isolated from a 22-year-old female diagnosed with influenza-like illness); this strain acquired E271K mutation during initial passage in RD cells (10), and A1/1348 (4310901348, isolated from a 36-year-old male diagnosed with common cold) were obtained from the National Institute of Public Health and the Environment (RIVM, The Netherlands). All strains were isolated in RD cells. EV-D68 B2/2042 was continued on RD cells (human rhabdomyosarcoma; ATCC, USA), while B2/947 and A1/1348 were cultured in HeLa cells (human adenocarcinoma; ATCC, USA). All cell lines were maintained in Eagle’s minimum essential medium (EMEM; Lonza, Switzerland) supplemented with l-glutamine (200 nM; Lonza, Switzerland), penicillin/streptomycin (100 U/mL each; Lonza, Switzerland), nonessential amino acids (ScienCell Research Laboratories, Canada), and fetal bovine serum (Sigma-Aldrich, Germany). The 50% tissue culture infective dose (TCID50) of virus was determined in RD cells using the Reed and Muench method (48). Viral stocks were sequenced prior to infection and B2/947 was confirmed to have the E271K mutation in VP1, while B2/2042 and A1/1348 did not have this strain-specific HSPG-binding mutation.

### Human airway epithelial (HAE) cultures.

HAE nasal MucilAir cultures derived from three individual donors (see Table 1 for donor information) were purchased from Epithelix Sàrl (Switzerland). The inserts were cultured, upon reception, for 1 week at an air-liquid interface before performing the infection experiments. The HAE inserts were cultured in MucilAir culture medium (Epithelix Sàrl, Switzerland) and the medium was refreshed every 2 to 3 days.

**TABLE 1:** Donor information for HAE cultures

Donor	Age	Sex	Race	Smoker?	Pathology
MD048401	55	Male	Caucasian	Nonsmoker	No pathology reported
MD0043601	46	Male	Caucasian	Nonsmoker	No pathology reported
MD019702	45	Male	Unknown	Unknown	No pathology reported

### Infection of HAE cultures.

All infection experiments were performed with one insert per condition with the three different donors considered biological replicates. Prior to infection, apical surfaces of the HAE cultures were washed thrice with 200 μL Hanks’ Balanced Salt Solution (HBSS; ThermoFisher Scientific, USA). HAE cultures were then inoculated with EV-D68 by adding 50 μL of virus stock (B2/947 6.1 logTCID50/mL, A1/1348 6.36 logTCID50/mL, and B2/2042 4.86 logTCID50/mL as determined on RD99 cells) to the apical side (as this is the physiological side of infection *in vivo*) and incubated for 2 h at 37°C, 5% CO_2_. After 2 h incubation, virus inoculum was discarded, and the apical surface was washed thrice with HBSS. The RD cell culture medium was added to the apical side for MOCK infection. For time point 0h, 100 μL of HBSS was added to the apical side and incubated for 10 min. After incubation, 100 μL of HBSS and 100 μL of culture medium was collected from the apical and basolateral sides, respectively. For the other time points, 100 μL of HBSS was first added to the apical side followed by a 10 min incubation prior to sample collection from the apical and basolateral sides. After each sample collection from the basolateral side 100 μL of fresh culture medium was added to keep the total volume at 600 μL per insert.

### Receptor blocking in HAE cultures.

Apical surfaces of the HAE cultures were washed thrice with 200 μL HBSS and the inserts were transferred to a new 24-well plate. For sialic acid blocking, 50 μL of neuraminidase solution (1:50 ratio in HBSS) was added to the apical side and incubated for 1 h. To ensure complete hydrolyses of sialic acid, neuraminidase from Arthrobacter ureafaciens (Roche diagnostics, Switzerland) and neuraminidase from Clostridium perfringens (New England BioLabs, USA) were used in equal proportion. For inserts that were not part of the sialic acid condition (untreated and heparin conditions), 50 μL of HBSS was added to the apical side and incubated for 1 h. In parallel, for blocking HSPG, virus stock was incubated with a working concentration of 5 mg/mL heparin sodium salt (H4784; Sigma-Aldrich, Germany) dissolved in phosphate-buffered saline (PBS; Sigma-Aldrich, Germany) or equal amount of PBS for untreated and neuraminidase conditions at 37°C, 5% CO_2_. After 1 h, inserts were washed once with 100 μL HBSS and inoculated with 50 μL of virus or virus/heparin solution. For MOCK inserts, HBBS or media/heparin solution was used depending on the condition. The virus was inoculated for 2 h and sample collection at time point 0 and 8h were performed as described earlier.

As complete receptor blocking would not be possible, we analyzed the fold change in viral copies between the 0 and 8 hpi, reflecting the first replication cycle for picornaviruses when the receptor blocking effect will be most prominent.

### Immunofluorescence staining and confocal microscopy of HAE cultures.

Inserts were washed thrice apically and basolaterally with HBBS at the end time point (72h for infection, 8h for receptor blocking). The inserts were then fixed with 4% formalin (Sigma-Aldrich, Germany) in PBS for 30 min at room temperature (RT). Membranes from the HAE inserts were then excised and submerged in 0.1% Triton X-100 for 15 min at RT to permeabilize the cells. Following permeabilization, membranes were treated with a solution containing 0.5% Tween20 (Sigma-Aldrich, Germany) and 10% bovine serum albumin (BSA; Sigma-Aldrich, Germany) in PBS and incubated overnight at 4°C to block nonspecific binding of antibodies in the subsequent steps. Between the fixation, permeabilization, and blocking steps, the membranes were washed thrice with PBS. Membranes were then cut into two equal pieces before treating the inserts with primary and secondary antibodies. All antibodies were diluted in a solution containing 0.5% Tween20 and 3% BSA dissolved in PBS. For primary antibody staining, membranes were incubated at 4°C overnight followed by washing thrice with 0.5% Tween20 in PBS. Secondary antibody staining was performed for 1 h at RT. The following primary antibodies were used in different combinations – mouse monoclonal β-tubulin-Cy3 (128K4872, Sigma-Aldrich, USA), mouse monoclonal mucin 5B (sc-393952, Santa Cruz Biotechnology, USA), goat polyclonal p63 (AF1916, R&D systems, USA), and rabbit polyclonal EV-D68 VP1 (GTX132313, GeneTex, USA). After secondary antibody staining, membranes were mounted on objective slides with ProLon Gold Antifade Mountant with DAPI (P36931, ThermoFisher Scientific, USA). Mounted membranes were imaged using Leica TCS SP8 X microscope (Leica Microsystems, Germany) and images were analyzed using Leica LAS X software (Leica Microsystems, Germany).

### Cerebral organoid cultures.

Human iPSCs, IMR90-4 (WiCell, USA) were cultured in mTesr^+^ medium (STEMCELL Technologies, Canada) on vitronectin (STEMCELL Technologies, Canada)-coated six-well plates and were passaged using ReLeSR (STEMCELL Technologies, Canada). Cerebral organoids were made from these iPSCs, using the cerebral organoid kit from STEMCELL Technologies. In short, 9,000 iPSCs were seeded per well in a round-bottom ultra-low-attachment plate (Corning, USA). Embryonic bodies (EBs) formed and were differentiated into cerebral organoids according to protocol and matured in a six-well plate while shaking on an orbital shaker (INFORS, Switzerland) at 66 rpm at 37°C and 5% CO_2_. With this protocol, four batches of organoids were made from the same iPSC line but with different passage numbers.

### Infection and receptor blocking of cerebral organoids.

Cerebral organoid infection was performed on one batch of 45-day-old organoids and three batches of 52-day-old organoids. Prior to infection, cerebral organoids were transferred to an ultra-low-attachment 96-well plate (Corning, USA) and organoid maturation medium (STEMCELL Technologies, Canada) was removed from the wells. For sialic acid blocking, 50 μL of neuraminidase solution (1:50 ratio in organoid maturation medium), all other organoids were incubated in 50 μL organoid maturation medium. After 1 h, organoids were washed once with 200 μL organoid maturation medium, medium was removed and organoids were inoculated with 50 μL of virus (B2/947 4.8 logTCID50/mL, A1/1348 7 logTCID50/mL or B2/2042 6.25 logTCID50/mL) that was preincubated at 37°C for 1 h with or without 5 mg/mL heparin sodium salt dissolved in phosphate-buffered saline. or virus pretreated with heparin. Inoculated cerebral organoids were then incubated for 1 h at 37°C, 5% CO_2_. Then, inoculum was removed, and the organoids were washed thrice with DMEM. After the third wash, the organoids were transferred to a 48-well plate, 500 μL of organoid maturation medium was added and the organoids were incubated at 37°C, 5% CO_2_. After 1 h (t0), 24, 48 and 72 hpi, the medium was sampled and refreshed with 500 μL maturation medium. Samples were stored at −80°C.

### EV-D68 detection by RT-qPCR and TCID50.

Twenty-five μL of collected culture medium was lysed and RNA was extracted using the MagNA Pure LC instrument (Roche Diagnostics, Switzerland) and the MagNA Pure LC Total Nucleic Acid isolation kit (03038505001, Roche Diagnostics, Switzerland) as per the manufacturer’s instructions. 40 μL of the 50 μL RNA eluate was reverse transcribed as described earlier (49). Five μL of cDNA was then used for RT-qPCR and analyzed using LightCycler 480 (Roche diagnostics, Switzerland) as per the manufacturer’s instructions with the EV-D68 primers (Forward: 5′-TGT TCC CAC GGT TGA AAA CAA -3′;Reverse: 5′-TGT CTA GCG TCT CAT GGT TTT CAC -3′; Biolegio, The Netherlands) and probes (probe 1: 6-FAM- ACC GCT ATA GTA CTT CG –MGB NFQ; Probe 2: 6-FAM- TCC GCT ATA GTA CTT CG –MGB NFQ; Biolegio, The Netherlands). For cerebral organoid medium samples, 25 μL of collected culture medium was lysed and RNA was extracted using the Purelink RNA minikit (Invitrogen) as per the manufacturer’s instructions. Forty μL of the 60 μL RNA eluate was reverse transcribed as described earlier (49). For TCID50, 25 μL of collected culture medium was serially diluted 1:10 in E8 medium. Fifty μL of diluted sample was added to 150 μL of RD99 cell suspension in quadruplicates in a 96-well plate and incubated at 37°C, 5% CO_2_. After 7 days, the plates were scored and the titer was calculated via the Reed Muench method.
