# Supplementary material for: Enterovirus D68 Infection in Human Primary Airway and Brain Organoids: No Additional Role for Heparan Sulfate Binding for Neurotropism
Source: Microbiol Spectr. 2022 Sep 26;10(5):e01694-22. doi: 10.1128/spectrum.01694-22 (PMC9603061; doi:10.1128/spectrum.01694-22)
Supplement: Supplemental file 1 — Supplemental material. Download spectrum.01694-22-s0001.pdf, PDF file, 0.7 MB [file spectrum.01694-22-s0001.pdf]

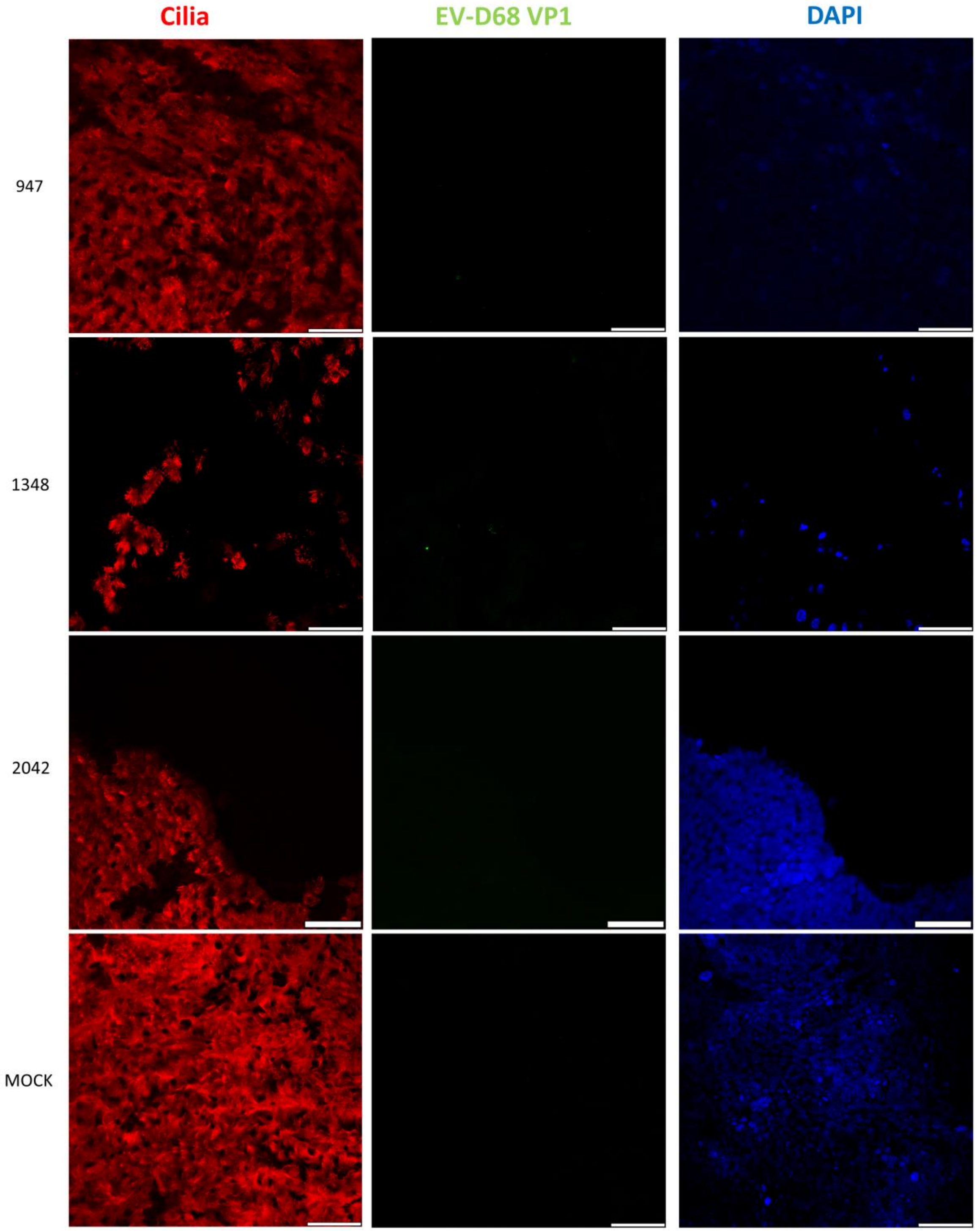

Supplementary figure 1.

Immunofluorescent staining of EV-D68 infection of HAE cultures 8 hpi under different conditions. Separate colour panels of Figure 3, with EV-D68 VP1 (green), ciliated cells (red) and nuclei (blue). Scale bars, 50 µm.

A

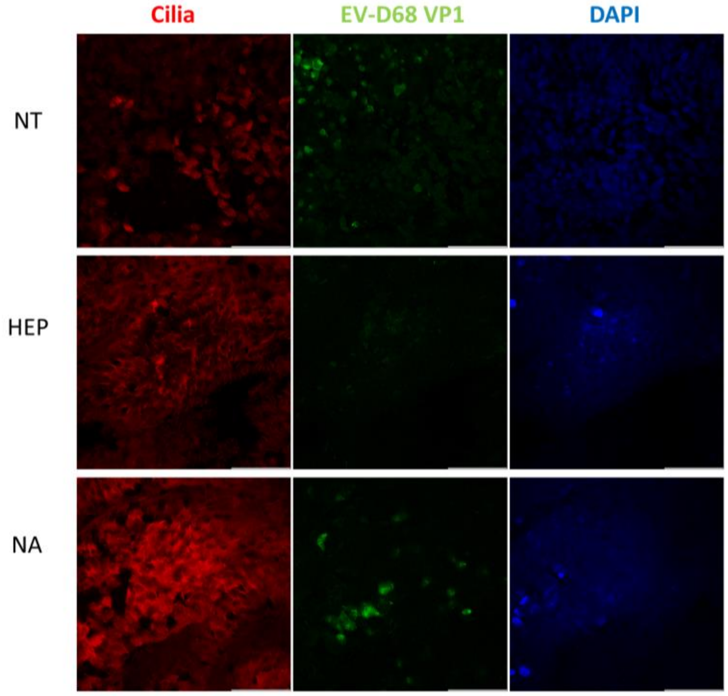

B

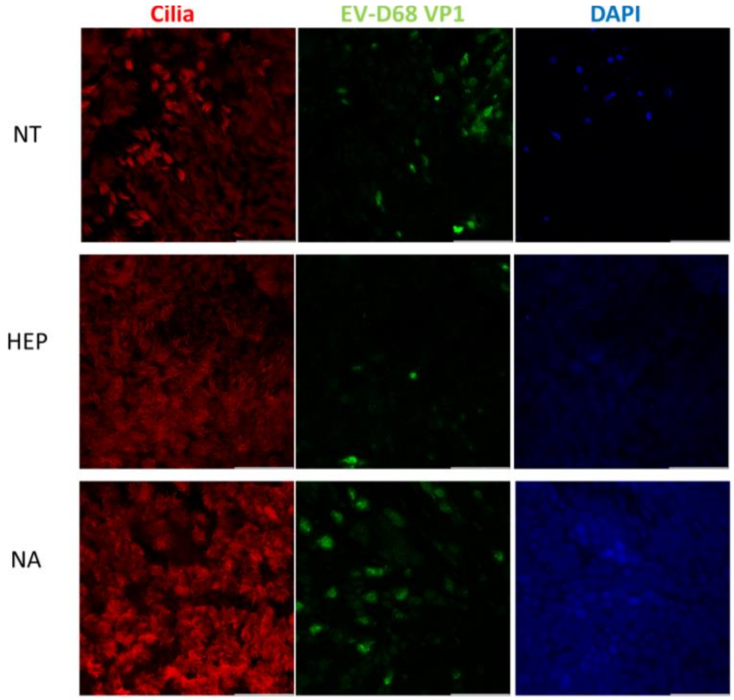

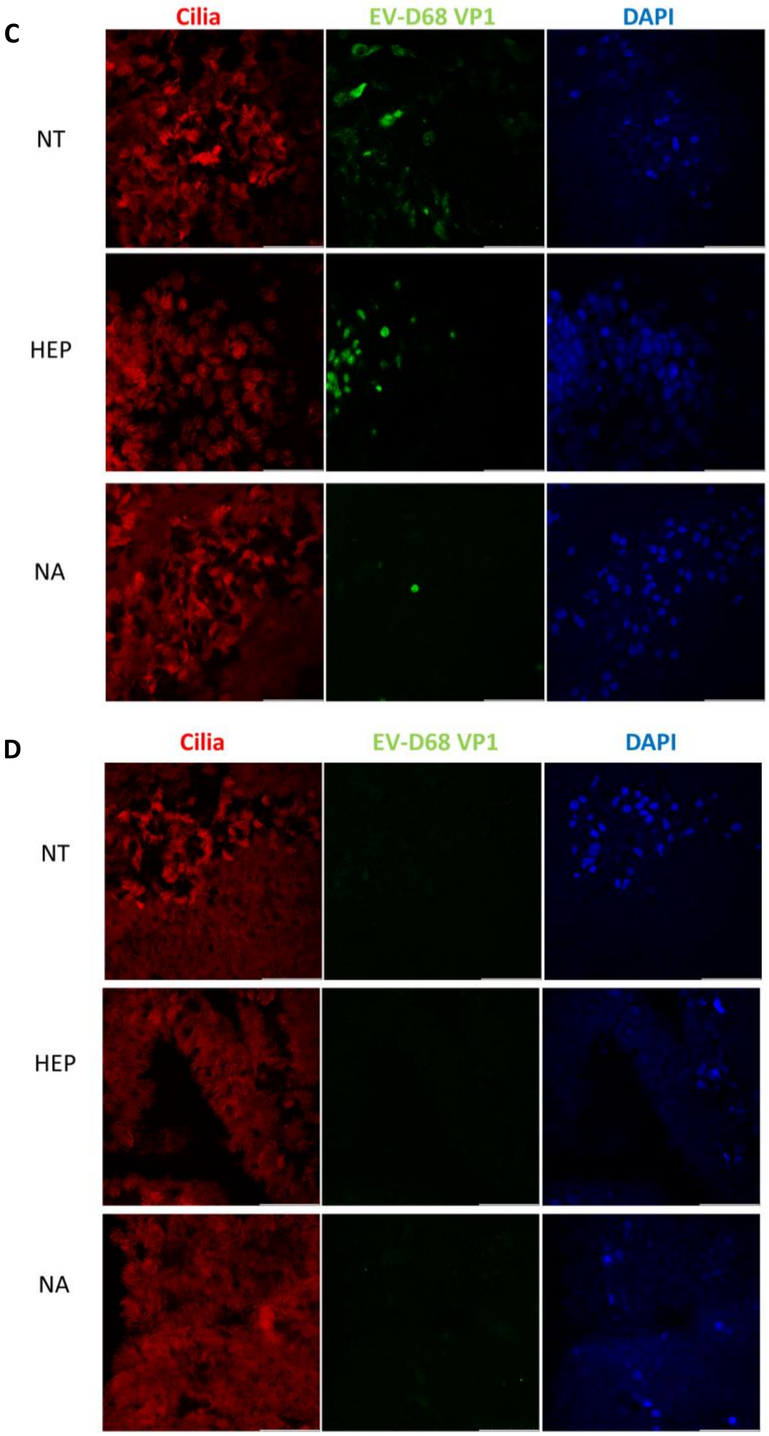

Supplementary figure 2.
Immunofluorescent staining of EV-D68 947 (A), 1348 (B), 2042 (C) and Mock (D) infection of HAE cultures 72hpi under different conditions, no treatment (NT), heparin (HEP) or neuraminidase (NA). Separate colour panels of of Figure 4, with EV-D68 VP1 (green), ciliated cells (red) and nuclei (blue). Scale bars, 50 µm.

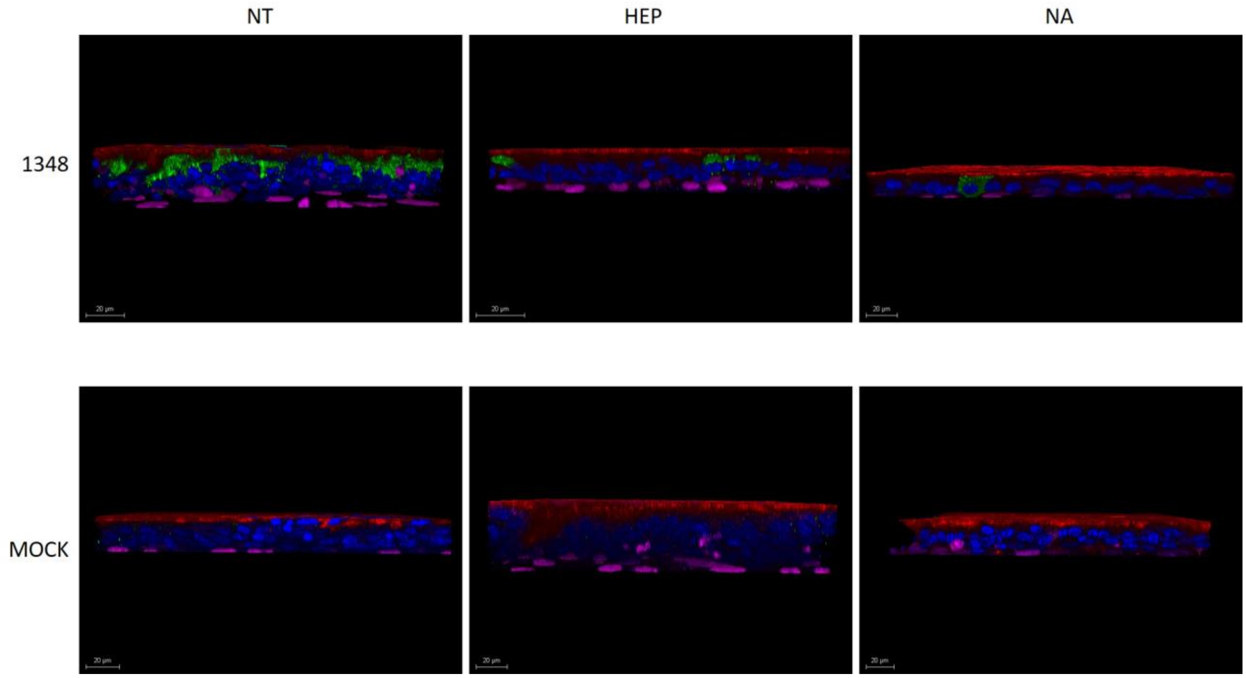

Supplementary figure 3.

3D side view of EV-D68 1348 infection and MOCK infection of HAE cultures 8 hpi under different conditions, no treatment (NT), heparin (HEP) or neuraminidase (NA). Legend: EV-D68 (green), ciliated cells (red), basal cells (purple), and nuclei (blue). Scale bars, 20 µm.
